# Tip Pressure on Semicircular Specimens in Tapping Mode Atomic Force Microscopy in Viscous Fluid Environments

**DOI:** 10.3390/s17102182

**Published:** 2017-09-22

**Authors:** Hua-Ju Shih, Ching-Liang Dai, Po-Jen Shih

**Affiliations:** 1 Institute of Applied Mechanics, National Taiwan University, No. 1, Sec. 4, Roosevelt Rd., Taipei 10617, Taiwan; d04543001@ntu.edu.tw; 2 Department of Mechanical Engineering, National Chung Hsing University, No. 145, Xingda Rd. South Dist., Taichung 40227, Taiwan; cldai@dragon.nchu.edu.tw; 3 Department of Civil and Environmental Engineering, National University of Kaohsiung, No. 700, Kaohsiung University Rd., Nanzih District, Kaohsiung 81148, Taiwan

**Keywords:** tapping mode, atomic force microscopy, pressure, vorticity, semi-analytical method

## Abstract

Tapping mode (TM) atomic force microscopy (AFM) in a liquid environment is widely used to measure the contours of biological specimens. The TM triggers the AFM probe approximately at the resonant frequencies and controls the tip such that it periodically touches the specimen along the scanning path. The AFM probe and its tip produce a hydrodynamic pressure on the probe itself and press the specimen. The tip to specimen size ratio is known to affect the measurement accuracy of AFM, however, few studies have focused on the hydrodynamic pressure caused by the effects of specimen size. Such pressure affects the contour distortion of the biological specimen. In this study, a semi-analytical method is employed for a semicircular specimen to analyze the vorticity and pressure distributions for specimens of various sizes and at various tip locations. Changes in pressure distribution, fluid spin motion, and specimen deformation are identified as the tip approaches the specimen. The results indicate the following: the specimen surface experiences the highest pressure when the specimen diameter equals the tip width; the vorticity between tip and specimen is complex when the tip is close to the specimen center line; and the specimen inflates when the tip is aligned with the specimen center line.

## 1. Introduction

To investigate nanoscale biological specimens—measuring their surface contours, for example—atomic force microscopy (AFM) is a superior technique to scanning electron microscopy (SEM) [1,2]. However, using AFM to observe specimens containing water or specimens immersed in a fluid environment can be problematic. The two basic AFM operation modes for probing a liquid environment are the contact mode (CM) [3] and tapping mode (TM). Compared with CM, TM is capable of scanning soft surfaces with certain contact forces. Although the force at the tip is weak in the TM, the vibrating tip body produces fluid pressure and fluid vorticity at the edges of the tip and of the specimen, thereby distorting the measurement results. Notably, this distortion of surface morphology is different from the geometric effect between tip and specimen in air [4]. Numerous studies have discussed probe hydrodynamics [1,5,6], which are known to affect a probe’s natural frequencies [1,7]; however, few studies have focused on the specimen pressure caused by tips [8]. The authors of the present study demonstrated that the liquid pressure varies with the tip geometry; furthermore, the maximum hydrodynamic pressure caused by a cone-shaped tip is approximately 0.5 Pa, and this can predeform a membrane by several nanometers before the tip taps its surface [9]. The tip movement direction was also shown to influence bubble deformation [10]. In this paper, we improve upon our previous analytical technique and employ it to study the hydrodynamic pressure on semicircular specimens of various diameters and using various tip positions to simulate scanning in a single-molecule force measurement. The semicircular specimen could also be employed in investigation of tip–nanobubble interaction.

In recent years, TM AFM has been used increasingly for bioscanning applications. TM AFM systems contain two key systems: the control and monitoring systems. The control system triggers the probe at the resonant frequency, measures the frequency and amplitude response, and adjusts the feedback loop to realize accurate measurements. The monitoring system provides the measured contours and elevation corrections to ensure an accurate scanning height. Elevation corrections comprise tip shape, specimen distortion, and scanning step corrections. Tip shape correction makes corrections for surfaces with a high aspect ratio; pressure corrects specimen distortion caused by external pressure from the probe and tip; and scanning step distance correction achieves the best performance in terms of scanning speed and point-to-point distance. Among these issues, few studies have focused on specimen distortion caused by fluid pressure. Kim et al. [11] found that when a standard grating sample was scanned using AFM in different fluids, the images were significantly distorted and the additional pressure between the cantilever and sample was strongly dependent on the kinematic viscosity of the fluid. Moreover, the additional pressure from the probe was dependent on the tip–specimen distance, probe width, tip shape, vibration frequency, and Reynolds number [5]. The maximum hydrodynamic pressure exerted by a tip is approximately 180 pN [12], which is 1.8 times larger than the peak force, the maximum contact force when the tip contacts the specimen. The hydrodynamic pressure is a periodic pressure produced when the tip taps the soft specimen and distorts it. When the topographies measured using TM AFM are compared with those measured using CM AFM, the height of a specimen as measured using TM AFM is always lower. For example, the height of an outer hexagonally packed intermediate layer as measured using TM AFM is approximately 25% smaller than that measured using CM AFM [13]. The mechanical stiffness of a protein and membrane is dependent on the probe’s approach speed [14]. Vichare et al. [15] studied cell geometry and noted that cell prestress may affect the measured stiffness. In addition, some AFM measurement results were used to identify biomarkers and thus distinguish diseases such as cancer. However, these mechanical properties may change under different approach pressures [16].

A beam oscillating with small amplitude in a liquid is described by the unsteady Navier–Stokes equation. Semi-analytical methods are widely used to solve the hydrodynamic pressure term in this equation. One such method was first used by Tuck [17] to solve the equation for a vibrating beam in an infinite liquid environment and then modified by Sader et al. [18,19,20] to solve the equation for a vibrating beam in a half plane. Green et al. [21,22] calculated added liquid mass by modifying the damping of the liquid and considering the normal and torsional modes of AFM, and thus the frequency shifts of the cantilever were predicted. In addition, the authors of the present study extended upon the studies of Sader et al. [18,19,20] to calculate the hydrodynamic pressure and vorticity on an AFM tip and on the surface of a plane-specimen [9]. The difficulty in extending the technique of Sader et al. was to consider the tip geometry in the boundary integration and the added kernels of integration. The present authors used mathematic terms to simplify the equations and applied coordinate transformations to increase the integration performance along the integral paths. The results demonstrated that the geometric curvature of the tip results in dramatic changes in vorticity and pressure at the tip. When the tip moves close to a specimen, the pressure on the specimen surface changes rapidly. Different tip shapes produce pressures on the plane specimen surface that can vary by a factor of 1.6.

In this paper, the authors discuss how pressure and vorticity affect a specimen. When an AFM tip comes close to a specimen, the geometric space between the tip and specimen leads vorticity field completed and results in specimen distortion. The results show that strong pressure is exerted when the tip width equals the specimen diameter, the vertical gap is less than 0.3 times the tip height, and the tip comes close to the center line of the specimen.

## 2. Methods

According to the authors’ previous study [9], tip curvature affects the vorticity around the tip and exerts high pressure on the specimen near the tip, when the specimen was assumed to be a planar surface. In this paper, the specimen is assumed to be semicircular. When the tip approaches the specimen, interaction between the tip and specimen result in complex vorticity and pressure.

### 2.1. Mathematical Model

The authors have modified the boundary integral method detailed in the study by Sader et al. [19] and added the contribution from the presence of both tip and specimen to the original model. We make the following five assumptions: (i) the system is two-dimensional; (ii) the amplitude of oscillation is small compared with the characteristic length of the cantilever; (iii) the fluid is incompressible; (iv) the surfaces of the tip, cantilever, and specimen are no-slip; and (v) the thickness of the cantilever is small compared with its width. The no-slip means the tangential fluid velocity on these surfaces is zero. In addition, the nonlinear convective inertial effects in the fluid were assumed to be negligible; therefore, only the linearized unsteady Navier-Stokes equation must be considered.

A beam oscillating with small amplitude in a liquid is described by the following Fourier-transformed unsteady Navier-Stokes equation:

(1) $$-i\omega \rho u=-\nabla P+\eta \nabla^{2}u$$

**Figure 1:**
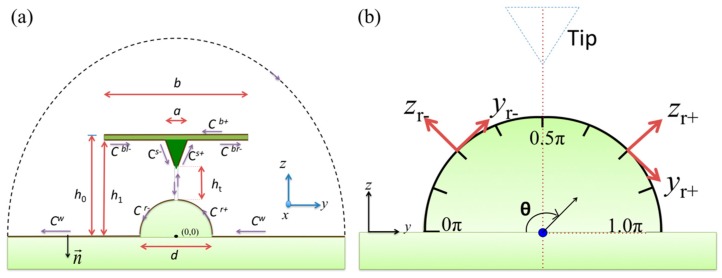
Two-dimensional cross-section of the probe, tip, and specimen: (**a**) integral path of the semi-analytical method in the global coordinate system and (**b**) local coordinate system relative to the semicircular specimen.

(2) ∇·u=0

where velocity vector, **u**(*y*,*z*|*ω*), is with two components (*v*,*w*); the pressure is denoted by *P*(*y*,*z*|*ω*); angular velocity by *ω*; density of the liquid by *ρ*; and viscosity of the liquid by *η*. The Fourier transform of a function with respect to time *t* is given by $\hat{x}\left(\omega \right)=\int_{-\infty }^{\infty }x\left(t\right)e^{i\omega t}dt$. A stream function ψ(y,z|ω) is introduced to describe the velocities v(y,z|ω)=∂ψ(y,z|ω)/∂z and w(y,z|ω)=−∂ψ(y,z|ω)/∂z in the *y*- and *z*- directions, respectively. For the unsteady Navier–Stokes equation, the application of Green’s integral theorem results in the following integral representation of the stream function:

(3) $$\begin{array}{c} \psi \left(y^{\prime },z^{\prime }|\omega \right)=\int_{C}[\psi \left(y,z|\omega \right)G_{n}\left(y,z|y^{\prime },z^{\prime }\right)-\psi_{n}\left(y,z|\omega \right)\Omega \left(y,z|\omega \right)\\ -\zeta \Psi_{n}\left(y,z|y^{\prime },z^{\prime }\right)+P\Psi_{l}\left(y,z|y^{\prime },z^{\prime }\right)/\eta ]dl \end{array}$$

where $G(y,z|y^{\prime },Z^{\prime })$ denotes the Green’s function of the Laplace equation; $\Omega (y,z|y^{\prime },z^{\prime })$ is the Green’s function of the Helmholtz equation; $\Psi (y,z|y^{\prime },z^{\prime })$ is the Green’s function of Equation (1); $\zeta (y,z|\omega )=-\nabla^{2}\psi (y,z|\omega )$ denotes the component of the vorticity in the *x*-direction; the increment *l* denotes differentiation along *C*; and *n* denotes differentiation normal to the boundary of *C* in out of the flow field direction. The detailed derivation of Equation (3) can be found in Tuck [17] and Sader et al. [19]. The integration path *C* in Equation (3) describes a cross-section of the system in fluid environment as shown in Figure 1a, and the two-dimensional free-space Green’s functions is appropriated to be used:

(4) $$G\left(y,z|y^{\prime },Z^{\prime }\right)=\left[\log R\right]/2\pi$$

(5) $$\Omega \left(y,z|y^{\prime },Z^{\prime }\right)=-K_{0}\left(\sigma R\right)/2\pi$$

(6) $$\Psi \left(y,z|y^{\prime },Z^{\prime }\right)=-\left[\log R+K_{0}\left(\sigma R\right)\right]/2\pi \sigma^{2}$$

where $R=\sqrt{{\left(y-y^{\prime }\right)}^{2}+{\left(z-z^{\prime }\right)}^{2}}$ and *K*_0_ is the modified third-order Bessel function. In the present model—which comprises the cantilever, tip, and specimen—the hydrodynamic loading from the tip on the specimen is the focus. Thus, the two unknown quantities in Equation (3) are *ζ*(*y*,*z*|*ω*) and *P*(*y*,*z*|*ω*). For a thin cantilever, the integration contour comprises four parts: the wall surface *C^w^*, the beam (beam top *C^b^*^+^ and beam bottom *C^b^*^−^), tip surface (tip right *C^s^*^+^ and tip left *C^s^*^−^), and the specimen surface (semicircle right *C*^s+^ and *C*^s−^). Because the surfaces of the tip and specimen are assumed to be no-slip, the terms *ψ* and *ψ_n_* are zero, and Equation (3) becomes:

(7) $$\begin{array}{c} \psi =\int_{C^{w}}\left[-\zeta^{w}\Psi_{n}+\frac{1}{\eta }P^{w}\Psi_{l}\right]dl+\int_{C^{b+}}\left[-\zeta^{b+}\Psi_{n}+\frac{1}{\eta }P^{b+}\Psi_{l}\right]dl\\ +\int_{C^{b-}}\left[-\zeta^{b-}\Psi_{n}+\frac{1}{\eta }P^{b-}\Psi_{l}\right]dl+\int_{C^{s+}}\left[-\zeta^{s+}\Psi_{n}+\frac{1}{\eta }P^{s+}\Psi_{l}\right]dl\\ +\int_{C^{s-}}\left[-\zeta^{s-}\Psi_{n}+\frac{1}{\eta }P^{s-}\Psi_{l}\right]dl+\int_{C^{r+}}\left[-\zeta^{r+}\Psi_{n}+\frac{1}{\eta }P^{r+}\Psi_{l}\right]dl\\ +\int_{C^{r-}}\left[-\zeta^{r-}\Psi_{n}+\frac{1}{\eta }P^{r-}\Psi_{l}\right]dl \end{array}$$

where (*ζ^w^*, *ζ^b+^*, *ζ^b^*^−^, *ζ^s^*^+^, *ζ^s^*^−^, *ζ^r+^*, and *ζ^r−^*) and (*P^w^*, *P^b^*^+^, *P^b^*^−^, *P^s^*^+^, *P^s^*^−^, *P^r+^*, and *P^r−^*) are the vorticity and pressure at the wall surface, beam top, beam bottom, tip right, tip left, specimen right, and specimen left, respectively. Note that the paths, except the specimen surface, are succeeded from the previous study [9]. The paths *C^w^*, *C^b^*^+^, and *C^b^*^−^ follow the global coordinate system (*y*, *z*), and the paths *C^s^*^+^, *C^s^*^−^, *C^r^*^+^, and *C^r^*^−^ should be transformed into the local coordinate systems (*y_s_*_+_, *z_s_*_+_), (*y_s_*_−_, *z_s_*_−_), (*y_r_*_+_, *z_r_*_+_), and (*y_r_*_−_, *z_r_*_−_) illustrated in Figure 1b. Therefore, *C^w^* extends from both *y* = −*∞* to *y* = −*d*/2 and *y* = *d*/2 to *y* = *∞*. On *C^w^*, we have the differentiations *dl* = −*dy*, ∂/∂*l =* −∂/∂*y*, and ∂/∂n = −∂/∂z. For the *C^r^*^+^ path, the transformation follows *y* = *y_r_*_+_cos β − *z_r+_*sin β and *z* = *y_r_*_+_cos β + *z_r_*_+_sin β. The integration along *y_r_*_+_ is from *y_r_*_+_ = 0 to *y_r_*_+_ = *r*, where *r* is the radius of the specimen. We have the differentiations *dl* = *dy_r_*_+_, ∂/∂*l* = −∂/∂*y_r_*_+_, and ∂/∂*n* = ∂/∂*z_r_*_+_ on *C^r^*^+^. For the *C^r^*^−^ path, the transformation follows *y* = *y_r_*_−_cos γ − *z_r−_*sin γ and *z* = *y_r_*_−_cos γ + *z_r_*_−_sin γ. The integration along *y_r_*_−_ is from *y_r_*_−_ = 0 to *y_r_*_−_ = *r*. We have the differentiations *dl* = *dy_r_*_−_, ∂/∂*l* = ∂/∂*y*_*r*−_, and ∂/∂*n* = −∂/∂*z_r_*_−_ on *C^r^*^+^. Using these relations, Equation (7) can be rewritten as:

(8) $$\begin{array}{l} \psi =\int_{-\infty }^{-d/2}\left[\zeta^{wl}\Psi_{z}-\frac{1}{\eta }P^{wl}\Psi_{y}\right]dy+\int_{d/2}^{\infty }\left[\zeta^{wr}\Psi_{z}-\frac{1}{\eta }P^{wr}\Psi_{y}\right]dy\\ +\int_{-b/2}^{b/2}\left[\zeta^{b+}\Psi_{z}-\frac{1}{\eta }P^{b+}\Psi_{y}\right]dy+\int_{-b/2}^{-a/b}\left[-\zeta^{bl-}\Psi_{z}+\frac{1}{\eta }P^{bl-}\Psi_{y}\right]dy\\ +\int_{a/2}^{b/2}\left[-\zeta^{br-}\Psi_{z}+\frac{1}{\eta }P^{br-}\Psi_{y}\right]dy+\int_{0}^{s}\left[-\zeta^{s+}\Psi_{s+}+\frac{1}{\eta }P^{s+}\Psi_{s+}\right]dy_{s+}\\ +\int_{0}^{s}\left[\zeta^{s-}\Psi_{z_{s-}}-\frac{1}{\eta }P^{s-}\Psi_{y_{s-}}\right]dy_{s-}+\int_{0}^{r}\left[-\zeta^{r+}\Psi_{r+}+\frac{1}{\eta }P^{r+}\Psi_{r+}\right]dy_{r+}\\ +\int_{0}^{r}\left[\zeta^{r-}\Psi_{r-}-\frac{1}{\eta }P^{r-}\Psi_{r-}\right]dy_{r-} \end{array}$$

in which *d* is the radius of specimen, *b* is the width of cantilever, *a* is the width of tip, *s* is the inclined length of the tip as shown in Figure 1. Differentiating Equation (8) with respect to *z’* and *y’* yields the velocity components *v* and *w*. This enables the evaluation of each integral equation at the specimen surface (*z’* = 0), beam surfaces (*z’* = *h*_0_ and *z’* = *h*_1_, where *h*_0_ and *h*_1_ are the locations of the beam top and beam bottom, respectively), tip surfaces ($z_{s+}^{\prime }=h_{t}+y_{s+}^{\prime }$ and $z_{s+}^{\prime }=h_{t}+y_{s+}^{\prime }\sin\theta$), and specimen surfaces ( $z_{r+}^{\prime }=y_{r+}^{\prime }$ and $z_{r-}^{\prime }=y_{r-}^{\prime }$). Thus, there are 18 coupled integral equations for the velocity components *v* and *w* and are listed in Appendix A. Although only consider the bending mode is considered, Equation (8) can be adapted to any AFM operating mode, such as the torsional mode.

It is assumed that the normal displacements on the surfaces and tip of the beam are the constant, *W*(*y’*,*z*|*ω*) = *W*_o_ and the displacement on the surface of the specimen is zero. There are 18 boundary conditions in the system. For the horizontal velocities on the surfaces, the following conditions must be satisfied:

(9) $$\begin{array}{l} v\left(y^{\prime },0|\omega \right)=0\;\mathrm{for}\;-\infty \leq y^{\prime }\leq d/2;\\ v\left(y^{\prime },0|\omega \right)=0\;\mathrm{for}\;d/2\leq y^{\prime }\leq \infty ;\\ v\left(y^{\prime },h_{0}|\omega \right)=0\;\mathrm{for}\;-b/2\leq y^{\prime }\leq b/2;\\ v\left(y^{\prime },h_{1}|\omega \right)=0\;\mathrm{for}\;-b/2\leq y^{\prime }\leq a/2;\\ v\left(y^{\prime },h_{1}|\omega \right)=0\;\mathrm{for}\;a/2\leq y^{\prime }\leq b/2;\\ v\left(y_{s-}^{\prime },h_{t}+y_{s-}^{\prime }\sin\alpha |\omega \right)=0\;\mathrm{for}\;-a/2\leq y^{\prime }\leq 0;\\ v\left(y_{s+}^{\prime },h_{t}+y_{s+}^{\prime }\sin\theta |\omega \right)=0\;\mathrm{for}\;0\leq y^{\prime }\leq a/2;\\ v\left(y_{r-}^{\prime },y_{r-}^{\prime }\sin\gamma |\omega \right)=0\;\mathrm{for}\;-c/2\leq y^{\prime }\leq 0;\\ v\left(y_{r+}^{\prime },y_{r+}^{\prime }\sin\beta |\omega \right)=0\;\mathrm{for}\;0\leq y^{\prime }\leq c/2. \end{array}$$

For the vertical velocities on the surfaces, the following conditions must be satisfied:

(10) $$\begin{array}{l} w\left(y^{\prime },0|\omega \right)=0\;\mathrm{for}\;-\infty \leq y^{\prime }\leq d/2;\\ w\left(y^{\prime },0|\omega \right)=0\;\mathrm{for}\;d/2\leq y^{\prime }\leq \infty ;\\ w\left(y^{\prime },h_{0}|\omega \right)=W\;\mathrm{for}\;-b/2\leq y^{\prime }\leq b/2;\\ w\left(y^{\prime },h_{1}|\omega \right)=W\;\mathrm{for}\;-b/2\leq y^{\prime }\leq a/2;\\ w\left(y^{\prime },h_{1}|\omega \right)=W\;\mathrm{for}\;a/2\leq y^{\prime }\leq b/2;\\ w\left(y_{s-}^{\prime },h_{t}+y_{s-}^{\prime }\sin\alpha |\omega \right)=W\;\mathrm{for}\;-a/2\leq y^{\prime }\leq 0;\\ w\left(y_{s+}^{\prime },h_{t}+y_{s+}^{\prime }\sin\theta |\omega \right)=W\;\mathrm{for}\;0\leq y^{\prime }\leq a/2;\\ w\left(y_{r-}^{\prime },y_{r-}^{\prime }\sin\gamma |\omega \right)=0\;\mathrm{for}\;-c/2\leq y^{\prime }\leq 0;\\ w\left(y_{r+}^{\prime },y_{r+}^{\prime }\sin\beta |\omega \right)=0\;\mathrm{for}\;0\leq y^{\prime }\leq c/2. \end{array}$$

Using Equations (A1) and (A2) in Appendix A, we simplify Equations (9) and (10) to a matrix notation:

(11) F=A·X

in which:

(12) $$A=\left[\begin{array}{ccc} A_{1,1} & \cdots & A_{1,18}\\ \vdots & \ddots & \vdots \\ A_{18,1} & \cdots & A_{18,18} \end{array}\right]$$

(13) $$X={\left\{\zeta^{wl},\;P^{wl},\;\zeta^{wr},\;P^{wr},\;\zeta^{b+},\;P^{b+},\;\zeta^{bl-},\;P^{bl-},\;\zeta^{br-},\;P^{br-},\;\zeta^{S+},\;P^{S+},\;\zeta^{S-},\;P^{S-},\;\zeta^{r+},\;P^{r+},\;\zeta^{r-},\;P^{r-}\right\}}^{T}$$

(14) $$F={\left\{0,\;0,\;0,\;0,\;0,\;W,\;0,\;W,\;0,\;W,\;0,\;W,\;0,\;W,\;0,\;0,\;0,\;0\right\}}^{T}$$

Following Tuck’s dimensionless definition [17], the dimensionless length is *ξ* = 2*y*/*b*, dimensionless pressure is $\bar{P=Pb/\left(2\eta Wo\right)}$, and dimensionless vorticity is $\bar{\zeta }=\zeta b/\left(2Wo\right)$ The vorticity is a spin field that describe the part of continuum in a small neighbourhood of the point, and it is diffused by the presence of viscosity. In many real flows, the viscosity is neglected by replacing with high Reynolds number, and the vorticity is also negligible except in small regions of space surrounding axes of the vortices. High vorticity implies strong circulation centres, and it could be found at corners or junctions between specimen, tip, and wall as discussed in this paper. Furthermore, the Reynolds number of the flow is defined as Re(*ω*) = *ρωb*^2^/4*η* to describe the relative importance of the linear inertial and viscous terms in the unsteady Navier-Stokes equation. According to the authors pervious study [9], the Reynold numbers of the tapping mode is in range of 746–1256 kHz (for 190–320 kHz). The large Reynold number help increasing the inertia force term rather than the viscosity term, and high triggering frequency of probe results in less difference of pressure distribution between tip geometries.

### 2.2. Numerical Calculation

Equation (12) shows a matrix with 18 × 18 elements, but if integral paths containing only the substrate plane and two planes of the probe are considered, it becomes a 6 × 6 matrix. Furthermore, ∆*P* = *P*^b+^ − *P*^b^^−^ is assumed to replace the two plane pressures, in accordance with [6], which enables the reduction of the matrix in Equation (12) to a 4 × 4 matrix:

(15) $$\left[\begin{array}{cc} \begin{array}{cc} B_{11} & B_{12}\\ B_{21} & B_{22} \end{array} & \begin{array}{cc} B_{13} & B_{14}\\ B_{23} & B_{24} \end{array}\\ \begin{array}{cc} B_{31} & B_{32}\\ B_{41} & B_{42} \end{array} & \begin{array}{cc} B_{33} & B_{34}\\ B_{43} & B_{44} \end{array} \end{array}\right]\left\{\begin{array}{c} \begin{array}{c} \zeta^{w}\\ P^{w} \end{array}\\ \begin{array}{c} \Delta \zeta^{b}\\ \Delta P^{b} \end{array} \end{array}\right\}=\left\{\begin{array}{c} 0\\ \begin{array}{c} 0\\ \begin{array}{c} 0\\ W \end{array} \end{array} \end{array}\right\}$$

*B*_11_, *B*_14_, *B*_22_, and *B*_24_ have the following analytical solutions:

(16) $$\begin{array}{c} B_{11}\left(Re,\;H,\;x\right)=\frac{-i}{xRe}+i\left|x\right|K_{1}\left(-i\left|x\right|\sqrt{iRe}\right)\times s_{-2,0}\left(-ix\sqrt{iRe}\right)\\ +xK_{0}\left(-i\left|x\right|\sqrt{iRe}\right)s_{-1,1}\left(x\sqrt{iRe}\right) \end{array}$$

(17) $$B_{14}\left(Re,\;H,\;x\right)=\frac{-i}{Re}\left(\frac{2H}{4H^{2}+x^{2}}+\frac{2Hi\sqrt{iRe}K_{1}(-i\sqrt{iRe}\sqrt{4H^{2}+x^{2}})}{\sqrt{4H^{2}+x^{2}}}\right)$$

(18) $$B_{22}\left(Re,\;H,\;x\right)=\frac{i}{Re}(\frac{1}{x}+sgn\left(x\right)i\sqrt{iRe}K_{1}\left(-i\left|x\right|\sqrt{iRe}\right)$$

(19) $$B_{24}\left(Re,\;H,\;x\right)=\frac{ix}{Re}\left(\frac{1}{4H^{2}+x^{2}}+\frac{i\sqrt{iRe}K_{1}(-i\sqrt{iRe}\sqrt{4H^{2}+x^{2}})}{\sqrt{4H^{2}+x^{2}}}\right)$$

where *K*_1_ is a modified third-order Bessel function and *s_u_*_,*v*_ are Lommel functions. Finding analytical solutions for the other elements in Equation (15) is difficult. Accordingly, the path transformation is as follows: *C^w^* = *C^wl^* + *C^r^*^−^ + *C^r^*^+^ + *C^wr^* and ∆*C^b^* = *C^b^*^+^ − (*C^bl^*^−^ + *C^r^*^−^ + *C^r^*^+^ + *C^br^*^−^). The relationship between matrices **A** and **B** is presented in Appendix B. This method is feasible only under the assumption that the slope of the tip and the radius of the semicircle approach zero. Fortunately, they have similar forms:

(20) $$A_{nm}=\int^{\text{​}}\Psi_{n,m}\left(\zeta ,z|\zeta_{k}^{\prime },z^{\prime }\right)d\zeta$$

where *m* = 1, 3, 5, …., 17 and *n* = 1, 3, 5, …., 17, and the primes indicate the mean observation for the midpoint of the integral piece $\zeta_{k}^{\prime }=0.5\left(\zeta_{k}+\zeta_{k+1}\right)$, k=0,1,…,N−1.

(21) $$A_{nm}=\int^{\text{​}}\Psi_{n,m}\left(\zeta ,y|\zeta_{k}^{\prime },y^{\prime }\right)d\zeta$$

where *m* = 2, 4, 6, …., 18 and *n* = 2, 4, 6, …., 18. Therefore, a Gaussian function is applied to discretize the total integration into pieces along the integral path (*C^wr^*, *C^wl^*, *C^r+^*, *C^r−^*, *C^s+^*, *C^s−^*, *C^br−^*, *C^bl−^*, and *C^b+^*) using the integral Gaussian points at ζ_j_ = −*L* cos(π *j*/*N* + 1), *j* = 1,2,3,…,*N* along length *L*. In the calculation, each path is first discretized into *N* = 200 Gaussian pieces, and then each piece is cut into 512 segments. Therefore, the number of integral pieces in one path is 102,400 and that in the total integration is more than 9 × 10^7^. The accuracy of the integration is controlled within 10^−5^. In our numerical tests, at least *N* > 120 × 128 = 15,360 pieces are required to achieve numerical convergence in each integral path within an accuracy of 10^−4^; otherwise, the Gaussian points are less dense at the ends of the path, which helps to fit the properties in *C^wr^* and *C^wl^* in far fields. In the numerical tests, the matrix **A** is reduced in size 4 × 4 to speed up the numerical calculation, because considerable time is required to solve inverse matrices. In the numerical calculation, the width of the tip, width of the probe, and height of the tip are kept constant and set at values obtained from the parameters of a normal AFM probe. The distance from the tip to the specimen is given by *h_t_* = 0.3*a*, and the diameter of the semicircle is *d*. The integral paths employed are as displayed in Figure 1a, and the normal and tangential vectors on the specimen surface are as defined in Figure 1b by *z_r+_*, *y_r+_*, *z_r−_*, and *y_r−_*; these are relative to the *C^r+^* and *C^r−^* paths.

## 3. Numerical Results

In this study, the geometric relationship between the specimen radius, tip width, and probe width are considered the crucial factors affecting the pressure and vorticity field. Four typical cases exist for this relationship: (1) the specimen is small: *d* < *a*; (2) the specimen is medium-sized: *a* < *d* < *b*; (3) the specimen is large: *b* < *d* < 2*b*; and (4) the specimen is very large: *d* >> 2*b*. For example, for a PPP-NCH-10 tip, the tip width is approximately 3.0 µm and the probe width is 22.5 µm. The following four cases thus exist: (1) *d* < 3 µm (e.g., suitable for coccus, rod-shaped bacteria, and bacillus), (2) 3 µm < *d* < 22.5 µm (lymphocytes, homosapiens, and monocytes), (3) 22.5 µm < *d* < 45 µm (plant and animal cells), and (4) *d* >> 45 µm (human egg cells and frog eggs).

**Figure 2:**
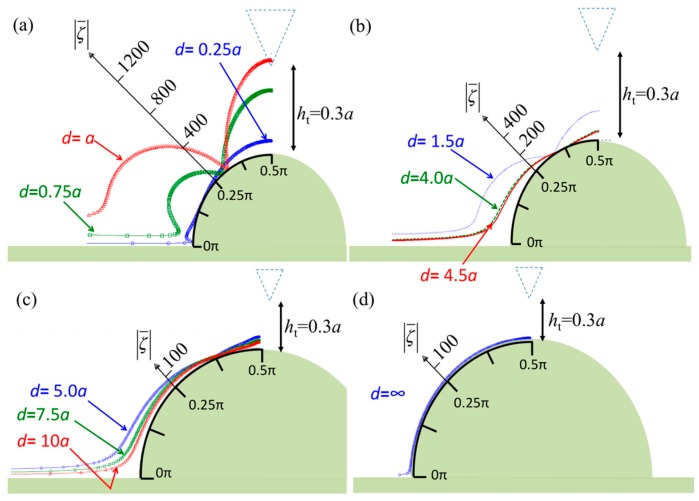
Normalized vorticities in the real part over the surface of the semicircular specimen when the diameter of the semicircle is (**a**) less than the tip width (*d* ≤ *a*); (**b**) between the tip width and probe width (*a* < *d* < *b*); (**c**) greater than the probe width (*b* ≤ *d* ≤ 2*b*); and (**d**) infinity.

Figure 2 displays the vorticity distribution along the specimen surface when the vibrating probe has a constant height, *h*_t_ = 0.3*a*, which is approximately 900 nm before tapping. Thus, the tip is close to the specimen before contact. In Figure 2, the polar coordinate system is illustrated to show the various vorticities with angles from *θ* = 0 to π/2 along the specimen surface. Furthermore, specimens with various ratios of tip width to radius are shown. For a small specimen (0.25*a* ≤ *d* ≤ *a*), the major vorticity is larger than 800 at the top and bottom edges of the specimen (Figure 2a). The strongest interaction occurs when the specimen diameter equals the tip width, with the strength of the interaction decreasing when the diameter is decreased. As the specimen size is increased, as illustrated in Figure 2b–d, the vorticity decreases. Interestingly, the vorticity is relatively large at the bottom corner and relatively small in the range of *θ* = 0.250π–0.375π.

**Figure 3:**
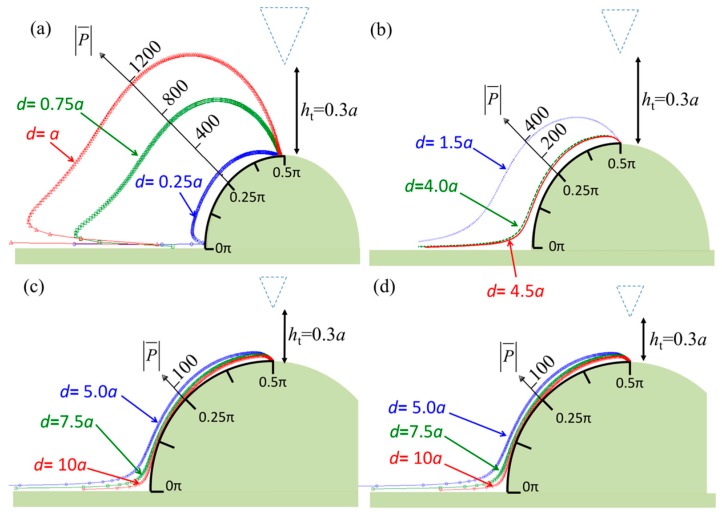
Normalized pressures in real part over the surface of the semicircular specimen when the diameter of the semicircle is (**a**) less than the tip width (*d* ≤ *a*); (**b**) between the tip width and probe width (*a* < *d* < *b*); (**c**) greater than the probe width (*b* ≤ *d* ≤ 2*b*); and (**d**) infinity.

Figure 3 shows the pressure distribution along the specimen surface when the vibrating probe is set at a constant height, *h*_t_ = 0.3*a*. The maximum pressure occurs when the specimen diameter is close to the tip width. The pressure pattern presented in Figure 3a is different from those in Figure 3b–d because the specimen is smaller than the tip width. The strong interaction between the tip and specimen causes a maximum pressure of more than 1200 Pa on the side of the specimen and zero pressure below the tip top and on the specimen surface. Figure 3b–d show similar pressure distributions, but the pressure decreases as the diameter is increased. Additionally, the patterns indicate low pressure along the range *θ* = 0.1π–0.4π when the specimen diameter is larger than *d* = 4*a*. The pressure is dramatically higher at the bottom corners because strong interference caused by the incident and reflective pressure waves can be expected at these locations.

**Figure 4:**
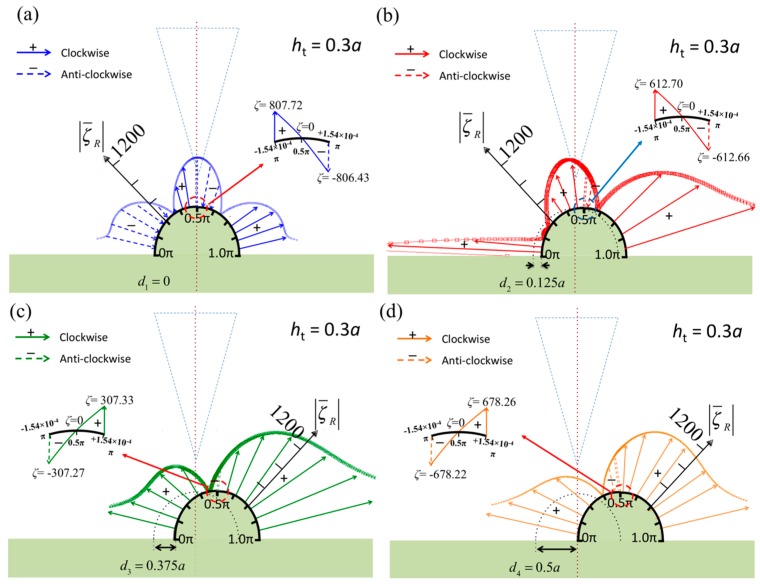
Normalized vorticities in real part over the surface of the semicircular specimen when the diameter of the semicircle *d* = *a*, *h*_t_ = 0.3*a*, and the tip is shifted to the left relative to the specimen center line. The shift magnitudes are (**a**) *d*_1_ = 0, (**b**) *d*_2_ = 0.125*a*, (**c**) *d*_3_ = 0.375*a*, and (**d**) *d*_4_ = 0.5*a*. Insets: the zero-vorticity points in the apical specimen.

**Figure 5:**
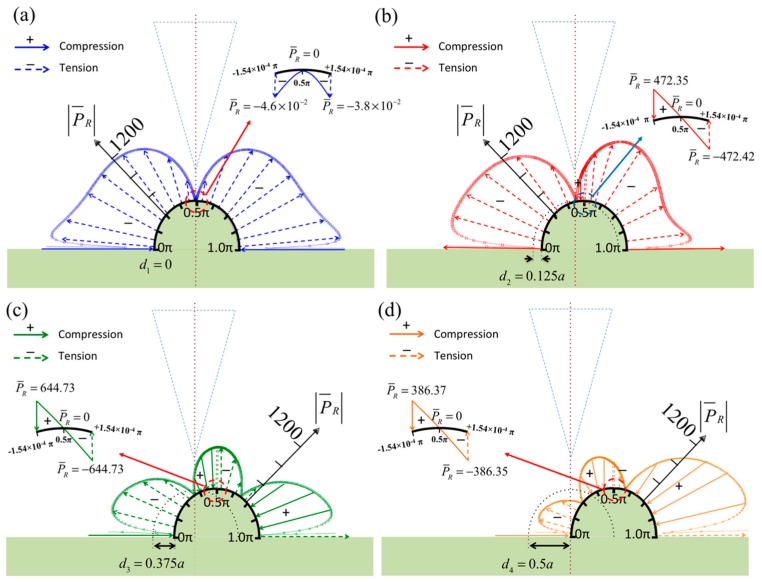
Normalized pressures in real part over the surface of the semicircular specimen when the diameter of the semicircle *d* = *a*, *h*_t_ = 0.3*a*, and the tip is shifted to the left relative to the specimen center line. The shift magnitudes are (**a**) *d*_1_ = 0, (**b**) *d*_2_ = 0.125*a*, (**c**) *d*_3_ = 0.375*a*, and (**d**) *d*_4_ = 0.5*a*. Insets: the zero-pressure points in the apical specimen.

In this analysis, the fundamental distributions of the vorticity and the pressure on the specimen surface when the tip center line is aligned with the specimen center line have been presented. Here, a moving tip is considered to simulate scanning at a constant height, and the horizontal shifts between the two center lines are set as *d_i_* = 0, 0.125*a*, 0.375*a*, and 0.5*a* (where *i* = 1, 2, 3, and 4). Figure 4 and Figure 5 display the vorticity and pressure distributions for these specific cases when the specimen diameter is *d* = *a*—the critical diameter—and the vertical gap between the tip and specimen is kept constant at *h*_t_ = 0.3*a*. The figures show that the tip center lines are shifted to the left relative to the specimen center line. A symmetric vorticity pattern is obtained when the tip is aligned at the center (Figure 4a); however, it should be noted that the vorticities at the sides have opposite directions, as indicated by the solid arrows (marked by “+”, clockwise) and dashed arrows (marked by “−”, anticlockwise). This is because an apical specimen separates the fluid field into two parts, as shown in the insets of Figure 4.

When the tip shifts to the left (Figure 4b–d), the vorticity increases on the right side of the specimen because the geometric gap between the tip and specimen is irregular, which results in complex vorticity. This phenomenon was also found for a planar specimen, as shown in Figure 2b of [5].

Figure 5a displays the symmetric pressure distribution on the specimen surface when the tip center line is aligned with the specimen center line. The normalized pressure is shown in the figure, and the signs of the real part of the pressure are marked to represent compression and tension. The pressure at the top center of the specimen is zero, as shown in the inset, and there are no phase changes on either side of the specimen; that is, both sides of the specimen are inflated or deflated together according to the AFM vibration frequency. When the tip is moved to the left by a distance *d*_2_ = 0.125*a* (Figure 5b), the pressure pattern changes, and the short area between the tip center line and specimen center line has opposite sign of pressure. This indicates that the pressure twists the surface in this area. Moreover, the twisted area increases when the tip is moved further to the left, as illustrated in Figure 5c,d. Interestingly, the left side of the specimen is always under negative pressure. However, on the right side, the area under negative pressure becomes small and the area under positive pressure becomes large.

**Figure 6:**
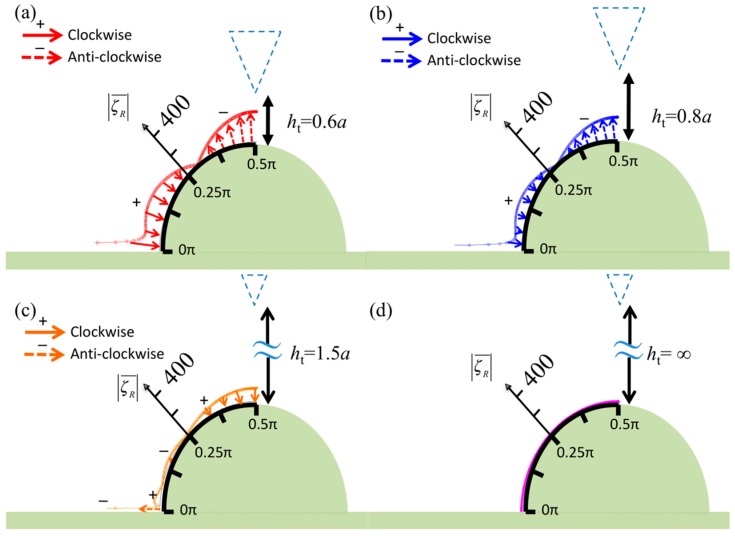
Normalized vorticities in real part over the surface of the semicircular specimen when the diameter of the semicircle *d* = *a* and the tip height is (**a**) *h*_t_ = 0.6*a*, (**b**) *h*_t_ = 0.8*a*, (**c**) *h*_t_ = 1.5*a*, and (**d**) *h*_t_ = ∞.

**Figure 7:**
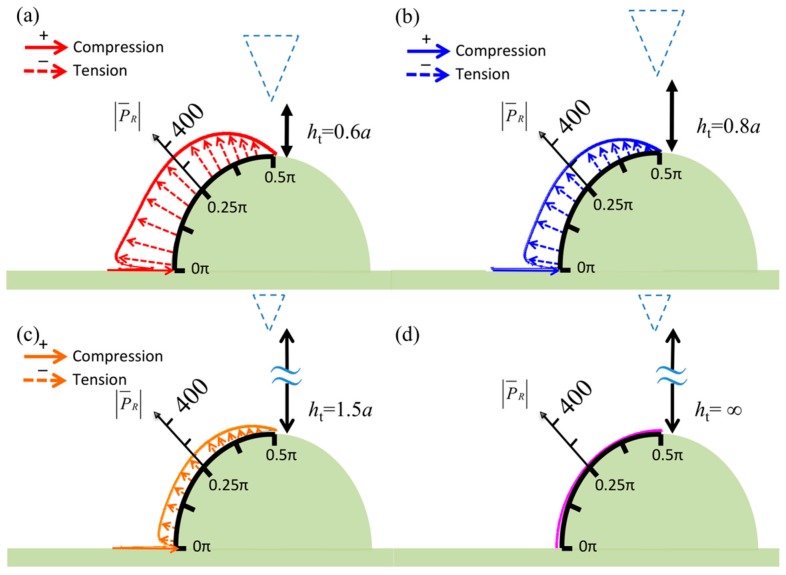
Normalized pressures in real part over the surface of the semicircular specimen when the diameter of the semicircle *d* = *a* and the tip height is (**a**) *h*_t_ = 0.6*a*, (**b**) *h*_t_ = 0.8*a*, (**c**) *h*_t_ = 1.5*a*, and (**d**) *h*_t_ = ∞.

Figure 6 shows the symmetric vorticity distribution on the specimen surface for various tip heights—*h*_t_ = 0.6*a*, 0.8*a*, 1.5*a*, and ∞—for which the case of *h*_t_ = 0.3*a* is presented in Figure 2a. The specimen diameter is *d* = *a* in all figures, and the tip center lines are aligned with the specimen center lines. The vorticity decreases when the tip height is increased. Furthermore, there is clearly a point in the range 0.25π–0.375π at which the vorticity changes direction. Figure 3a and Figure 7 display the pressure distribution on the specimen surface for various tip heights—*h*_t_ = 0.6*a*, 0.8*a*, 1.5*a*, and ∞, and 0.3*a*, respectively. The pressure increases to $\bar{P}=1200$ when the tip height is decreased to 0.3*a*. In the figures, *W* in Equation (10) is assumed to be +1 (upward in *z*-direction), and thus, the negative sign represents compression. This means that the removal of the tip allows the specimen to inflate, whereas the specimen deflates as the tip approaches.

## 4. Discussion

Generally, for a probe with a stiffness of 0.25 N/m in air, the force exerted by a typical tip on a specimen is 10–100 nN [23] (the vibrating amplitude is typically 40–400 nm). For tip measurement in a fluid environment, the probe stiffness is 0.095 N/m, and the tip force exerted on the specimen is approximately 0.6 nN (maximum) when the tip–sample separation is 200 nm [8]. Thus, the local force exerted by the tip is 99.4% lower for a liquid environment compared with an air environment. According to the result shown in Figure 3a, the pressure distribution $\bar{P}$ results in a pressure evaluated through the following calculation. Assuming *W*_0_ = −10 μm/s, *a* = 3 μm, *b* = 50 μm, and *η* = 1 × 10^−3^, the equation $P=2\eta W_{0}\bar{P}/b$ can be used and we let $\bar{P}=1200$; this obtains *P* = 4.8 × 10^−2^ Pa. For example, for a leukemia lymphoid cell [24], the probe stiffness is 0.02–0.08 kPa, and the specimen diameter is 6.8–7.3 µm. The strain is 6 × 10^−4^ to 2.4 × 10^−3^, and the associated deformation is 4.08–17.5 nm. Integrating the real part of the pressure along the left side of the semicircle, $\int_{0^{+}}^{\pi /2}\eta W_{o}Real\left(\bar{P}\right)\cos\theta \;a/b\;d\theta$, obtains an integral result of 0.723 nN (horizontal direction). According to this number, the semicircle experienced two side forces, and both sides were under tension. For the vertical direction, the result was 0.857 nN, which is obtained using the integral equation $\int_{0^{+}}^{\pi^{\_}}\eta W_{o}Real\left(\bar{P}\right)\sin\theta \;a/b\;d\theta$. Thus, the total hydrodynamic force is 1.22 nN; this force is larger than the force exerted by the tip. The tip force exerts a large pressure over a small area and bends the local specimen; by contrast, the hydrodynamic pressure is applied to the surface, and it inflates or deflates the specimen.

**Figure 8:**
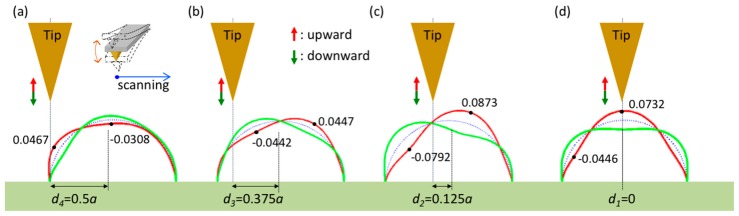
Vibrating amplitude of the specimen as the scanning tip approaches from a distance to the tip center (values are normalized by the factor $[\pi^{4}\eta W_{0}\bar{P}d^{4}/\left(8bEI)\right]$.

Regarding the vorticity, the zero-vorticity points at the surface separate the fluid into parts with antisymmetric spinning motions. In Figure 2a and Figure 6, the zero-vorticity points occur in the range 0.25π–0.375π and at the apical points (θ=0.5π). Therefore, there are three zero-vorticity points. In Figure 4b, however, there are four such points, and two such points are present in Figure 4c,d. As a result, the tip near the specimen central line causes complex vorticity and twists the local surface because the two apical points are too close and their relative motion induces a local spinning motion. This is also seen in the pressure distribution over the range 0.375π–0.625π in Figure 5b. Moreover, as displayed in Figure 5, complex pressure is exerted on the specimen when the tip approaches it or withdraws. Furthermore, the pressure distributions represent the potential surface distortion. Pressures marked with negative signs indicate inflation as the tip withdraws (upward); otherwise, the surface deflates as the tip approaches (downward). When the tip moves close to the specimen from a distance, as shown in Figure 5a–d, the deflation on the left side (tip side) increases. Furthermore, the right side first inflates and then decreases in size. Finally, the deflation on the left side increases. Figure 8 shows the tip approaching and withdrawing from the specimen and the corresponding specimen deformation. Notice that the tapping mode is mainly in vertical motion (approaching and withdrawing) and next in lateral motion (scanning) at high level. The scanning path of tapping mode AFM is like a long rectangular wave function; therefore, the vertical velocity of the probe affects pressure on specimen more than lateral velocity of the probe. Thus, there are four relative positions discussed in Figure 8.

The deformation is calculated by applying curved beam theory using the pressure loadings in Figure 5 and a uniform loading simulating the inner pressure of the cell. In addition, the total area of the specimen is assumed to be conserved in the calculation. According to the definition of the pressure, $P=2\eta W_{0}\bar{P}/b$ and the normalized displacement, $\bar{w}=[\pi^{4}\eta W_{0}\bar{P}d^{4}/\left(8bEI\right)]$, in Figure 8, the pressure *P* is proportional to the vertical velocity, *W*_0_. It means fast velocity enhances pressure on the specimen, and that results in large amplitude (vibration). Furthermore, this conclusion also could be found in the experiment [14]: Muller studied the relationship between vertical loading rate (velocity) and mechanical stiffness (relative to the reaction pressure). Their result shows that the mechanical stiffness increases with the loading rate (from 0.3 increasing to 3.2 N/m), and that indicates the fast loading results in large reaction from liquid pressure. Then, this pressure applies to the specimen.

The limitations of the semi-analytical model used herein are the assumptions made: a two-dimensional model, small amplitude of tip oscillation, incompressible fluid, and no-slip boundary condition. In addition, the authors present the results for *h*_t_ = 0.3*a* as the minimum tip height because this height is sufficient to represent the pressure distribution at low altitude. When the tip height is less than *h*_t_ = 0.3*a*, the local pressure between the tip and apical specimen becomes complex; however, the average pressure increases slightly, as illustrated in Figure 6 of [9], a study that focuses on global tip effects on a specimen instead of deformation in the local area. The surface contours of the scanning results of the specimen by AFM is drawn as array matrices, and many references [9,14,15] indicate the levels of the specimen are underestimation, especially for the soft bio-specimen scanned by the contact mode or the tapping mode in viscous environment. Thus, a feedback system which helps recover the scanning levels of the specimen based on the hydrodynamic pressure distribution is required to improve the accuracy of the scanning probe microscope.

The Reynolds number for AFM system is quite low (Re = 746~1256) obtained by the equation Re(ω)=ρωb2/4η. The Reynolds numbers here are below the reference value of the turbulence (Re = 2300). However, the probe vibrates fast in vertical direction and scans in horizontal direction, and the vorticity field changes with tip-specimen position. Those result in different vorticity patterns. Furthermore, the complicated boundary condition and high frequency vibration lead dramatic changes of vorticity and result in turbulence. They could be observed around the geometric corners, such as corner at the tip-specimen and at the specimen-wall. On the other hand, the gaps between the probe and the wall are narrow and small (*h_t_* = 5 μm, *h*_1_ = 15 μm). That leads the surface tension effect larger than the body force effect. The fluid transmits hydrodynamic pressure from probe to specimen through diffusive motion; and it’s the reason that the patterns of the vorticity are weakly relative to the patterns of the pressure as demonstrated above.

## 5. Conclusions

For a TM AFM probe, a fluid environment affects not only the shift in the resonant frequency of the probe but also the additional pressure on the specimen. The authors use a semi-analytical method to analyze the pressure and vorticity caused by a vibrating tip on a specimen surface. The results show that the vorticity and pressure change rapidly in the geometric space between the tip and specimen. Moreover, the tip exerts the highest pressure on the specimen when the specimen diameter equals the tip width, and the apical surface of the specimen twists rapidly when the tip center line is close to the specimen center line. Furthermore, the specimen surface inflates and deflates with the tip vibration and scanning. The authors calculate that the maximum deformation of a leukemia lymphoid cell surface will be 4.08–17.50 nm and that a hydrodynamic force of 1.22 nN will be exerted on both sides of the cell.

## Appendix A

For the observation points located at (*Y*, *Z*), the velocities in the *y*- and *z*-directions are as follows:

(A1) v(Y,Z|ω)=∫∞c[ζw+Ψzz′(y,0|y′,Z)−1ηpw+Ψyz′(y,0|y′,Z)]dy+∫−∞−c[ζw−Ψzz′(y,0|y′,Z)−1ηpw−Ψyz′(y,0|y′,Z)]dy+∫0r[−ζr+Ψzr+zr+′(yr+,0|yr+′,0)+1ηpr+Ψyr+zr+′(yr+,0|yr+′,0)]dzr+′×cosβ+∫0r[ζr+Ψzr+yr+′(yr+,0|yr+′,0)−1ηpr+Ψyr+yr+′(yr+,0|yr+',0)]dyr+′×(−sinβ)+∫0r[ζr−Ψzr−zr−′(yr−,0|yr−′,0)−1ηpr−Ψyr−zr−′(yr−,0|yr−′,0)]dzr−′×cosγ+∫0r[−ζr−Ψzr−yr−′(yr−,0|yr−′,0)+1ηpr−Ψyr−yr−′(yr−,0|yr−′,0)]dyr−′×(−sinγ)+∫−b2b2[ζb+Ψzz′(y,h0|y′,Z)−1ηpb+Ψyz′(y,h0|y′,Z)]dy+∫−b2−a2[−ζbl−Ψzz′(y,h1|y′,Z)+1ηpbl−Ψyz′(y,h1|y′,Z)]dy+∫a2b2[−ζbr−Ψzz′(y,h1|y′,Z)+1ηpbr−Ψyz′(y,h1|y′,Z)]dy+∫0s[−ζs+Ψzs+zs+′(ys+,0|ys+′,0)+1ηps+Ψys+zs+′(ys+,0|ys+′,0)]dzs+′×cosθ+∫0s[ζs+Ψzs+ys+′(ys+,0|ys+′,0)−1ηps+Ψys+ys+′(ys+,0|ys+′,0)]dys+′×(−sinθ)+∫0s[ζs−Ψzs−zs−′(ys−,0|ys−′,0)−1ηps−Ψys−zs−′(ys−,0|ys−′,0)]dzs−′×cosα+∫0s[−ζs−Ψzs−ys−′(ys−,0|ys−′,0)+1ηps−Ψys−ys−′(ys−,0|ys−′,0)]dys−′×(−sinα),

and:

(A2) w(Y,Z|ω)=∫∞c[−ζw+Ψzz′(y,0|y′,Z)+1ηpw+Ψyz′(y,0|y′,Z)]dy+∫−∞−c[−ζw−Ψzz′(y,0|y′,Z)+1ηpw−Ψyz′(y,0|y′,Z)]dy+∫0r[−ζr+Ψzr+zr+′(yr+,0|yr+′,0)+1ηpr+Ψyr+zr+′(yr+,0|yr+′,0)]dzr+′×cosβ+∫0r[ζr+Ψzr+yr+′(yr+,0|yr+′,0)−1ηpr+Ψyr+yr+′(yr+,0|yr+′,0)]dyr+′×(−sinβ)+∫0r[ζr−Ψzr−zr−′(yr−,0|yr−′,0)−1ηpr−Ψyr−zr−′(yr−,0|yr−′,0)]dzr−′×cosγ+∫0r[−ζr−Ψzr−yr−′(yr−,0|yr−′,0)+1ηpr−Ψyr−yr−′(yr−,0|yr−′,0)]dyr−′×(−sinγ)+∫−b2b2[−ζb+Ψzz′(y,h0|y′,Z)+1ηpb+Ψyz′(y,h0|y′,Z)]dy+∫−b2−a2[ζbl−Ψzz′(y,h1|y′,Z)−1ηpbl−Ψyz′(y,h1|y′,Z)]dy+∫a2b2[ζbr−Ψzz′(y,h1|y′,Z)−1ηpbr−Ψyz′(y,h1|y′,Z)]dy+∫0s[−ζs+Ψzs+zs+′(ys+,0|ys+′,0)+1ηps+Ψys+zs+′(ys+,0|ys+′,0)]dzs+′×cosθ+∫0s[ζs+Ψzs+ys+′(ys+,0|ys+′,0)−1ηps+Ψys+ys+′(ys+,0|ys+′,0)]dys+′×(−sinθ)+∫0s[ζs−Ψzs−zs−′(ys−,0|ys−′,0)−1ηps−Ψys−zs−′(ys−,0|ys−′,0)]dzs−′×cosα+∫0s[−ζs−Ψzs−ys−′(ys−,0|ys−′,0)+1ηps−Ψys−ys−′(ys−,0|ys−′,0)]dys−′×(−sinα),

**Table A1:** The variables of observation point (*Y*, *Z*) at each integral path.

	Wall		Specimen		Beam			Tip	
	*C^w+^*	*C^w−^*	*C^r+^*	*C^r−^*	*C^b+^*	*C^bl−^*	*C^br−^*	*C^s+^*	*C^s−^*
*Y*	*y*′	*y*′	$y_{r+}^{\prime }$	$y_{r-}^{\prime }$	*y’*	*y’*	*y’*	$y_{s+}^{\prime }$	$y_{s-}^{\prime }$
*Z*	0	0	$y_{r+}^{\prime }$×sin *β*	$y_{r-}^{\prime }$×sin (*γ*-90°)	*h*_0_	*h*_1_	*h*_1_	$y_{s+}^{\prime }$×sin *θ*	$y_{s-}^{\prime }$×sin (*α*-90°)

## Appendix B

(A3) $$B_{1,1}=\left(A_{1,1}+A_{3,1}+A_{15,1}+A_{17,1}\right)+\left(A_{1,3}+A_{3,3}+A_{15,3}+A_{17,3}\right)+\left(A_{1,15}+A_{3,15}+A_{15,15}+A_{17,15}\right)+\left(A_{1,17}+A_{3,17}+A_{15,17}+A_{17,17}\right)$$

(A4) $$B_{1,2}=(A_{1,2}+A_{3,2}+A_{15,2}+A_{17,2})+(A_{1,4}+A_{3,4}+A_{15,4}+A_{17,4})+(A_{1,16}+A_{3,16}+A_{15,16}+A_{17,16})+(A_{1,18}+A_{3,18}+A_{15,18}+A_{17,18})$$

(A5) $$B_{2,1}=(A_{2,1}+A_{4,1}+A_{16,1}+A_{18,1})+(A_{2,3}+A_{4,3}+A_{16,3}+A_{18,3})+(A_{2,15}+A_{4,15}+A_{16,15}+A_{18,15})+(A_{2,17}+A_{4,17}+A_{16,17}+A_{18,17})$$

(A6) $$B_{2,2}=(A_{2,2}+A_{4,2}+A_{16,2}+A_{18,2})+(A_{2,4}+A_{4,4}+A_{16,4}+A_{18,4})+(A_{2,16}+A_{4,16}+A_{16,16}+A_{18,16})+(A_{2,18}+A_{4,18}+A_{16,18}+A_{18,18})$$

(A7) $$\begin{array}{ll} B_{3,1} & =A_{5,1}-\left(A_{7,1}+A_{9,1}+A_{11,1}+A_{13,1}\right)+A_{5,3}-\left(A_{7,3}+A_{9,3}+A_{11,3}+A_{13,3}\right)\\ & +A_{5,15}-\left(A_{7,15}+A_{9,15}+A_{11,15}+A_{13,15}\right)+A_{5,17}-\left(A_{7,17}+A_{9,17}+A_{11,17}+A_{13,17}\right) \end{array}$$

(A8) $$\begin{array}{ll} B_{4,1} & =A_{6,1}-\left(A_{8,1}+A_{10,1}+A_{12,1}+A_{14,1}\right)+A_{6,3}-\left(A_{8,3}+A_{10,3}+A_{12,3}+A_{14,3}\right)\\ & +A_{6,15}-\left(A_{8,15}+A_{10,15}+A_{12,15}+A_{14,15}\right)+A_{6,17}-\left(A_{8,17}+A_{10,17}+A_{12,17}+A_{14,17}\right) \end{array}$$

(A9) $$\begin{array}{ll} B_{3,2} & =A_{5,2}-\left(A_{7,2}+A_{9,2}+A_{11,2}+A_{13,2}\right)+A_{5,4}-\left(A_{7,4}+A_{9,4}+A_{11,4}+A_{13,4}\right)\\ & +A_{5,16}-\left(A_{7,16}+A_{9,16}+A_{11,16}+A_{13,16}\right)+A_{5,18}-\left(A_{7,18}+A_{9,18}+A_{11,18}+A_{13,18}\right) \end{array}$$

(A10) $$\begin{array}{ll} B_{1,3} & =(A_{1,5}+A_{3,5}+A_{15,5}+A_{17,5})-(A_{1,7}+A_{3,7}+A_{15,7}+A_{17,7})-(A_{1,9}+A_{3,9}+A_{15,9}+A_{17,9})\\ & -(A_{1,11}+A_{3,11}+A_{15,11}+A_{17,11})-(A_{1,13}+A_{3,13}+A_{15,13}+A_{17,13}) \end{array}$$

(A11) $$\begin{array}{ll} B_{4,2} & =A_{6,2}-\left(A_{8,2}+A_{10,2}+A_{12,2}+A_{14,2}\right)+A_{6,4}-\left(A_{8,4}+A_{10,4}+A_{12,4}+A_{14,4}\right)\\ & +A_{6,16}-\left(A_{8,16}+A_{10,16}+A_{12,16}+A_{14,16}\right)+A_{6,18}-\left(A_{8,18}+A_{10,18}+A_{12,18}+A_{14,18}\right) \end{array}$$

(A12) $$\begin{array}{ll} B_{2,3} & =(A_{2,5}+A_{4,5}+A_{16,5}+A_{18,5})-(A_{2,7}+A_{4,7}+A_{16,7}+A_{18,7})\\ & -(A_{2,9}+A_{4,9}+A_{16,9}+A_{18,9})-(A_{2,11}+A_{4,11}+A_{16,11}+A_{18,11})-(A_{2,13}+A_{4,13}+A_{16,13}+A_{18,13}) \end{array}$$

(A13) $$\begin{array}{ll} B_{1,4} & =(A_{1,6}+A_{3,6}+A_{15,6}+A_{17,6})-(A_{1,8}+A_{3,8}+A_{15,8}+A_{17,8})\\ & -(A_{1,10}+A_{3,10}+A_{15,10}+A_{17,10})-(A_{1,12}+A_{3,12}+A_{15,12}+A_{17,12})-(A_{1,14}+A_{3,14}+A_{15,14}+A_{17,14}) \end{array}$$

(A14) $$\begin{array}{ll} B_{2,4} & =(A_{2,6}+A_{4,6}+A_{16,6}+A_{18,6})-(A_{2,8}+A_{4,8}+A_{16,8}+A_{18,8})\\ & -(A_{2,10}+A_{4,10}+A_{16,10}+A_{18,10})-(A_{2,12}+A_{4,12}+A_{16,12}+A_{18,12})-(A_{2,14}+A_{4,14}+A_{16,14}+A_{18,14}) \end{array}$$

(A15) $$\begin{array}{ll} B_{3,3} & =\left[A_{5,5}-\left(A_{7,5}+A_{9,5}+A_{11,5}+A_{13,5}\right)\right]-\left[A_{5,7}-\left(A_{7,7}+A_{9,7}+A_{11,7}+A_{13,7}\right)\right]-\left[A_{5,9}-\left(A_{7,9}+A_{9,9}+A_{11,9}+A_{13,9}\right)\right]\\ & -\left[A_{5,11}-\left(A_{7,11}+A_{9,11}+A_{11,11}+A_{13,11}\right)\right]-\left[A_{5,13}-\left(A_{7,13}+A_{9,13}+A_{11,13}+A_{13,13}\right)\right] \end{array}$$

(A16) $$\begin{array}{ll} B_{4,3} & =\left[A_{6,5}-\left(A_{8,5}+A_{10,5}+A_{12,5}+A_{14,5}\right)\right]-\left[A_{6,7}-\left(A_{8,7}+A_{10,7}+A_{12,7}+A_{14,7}\right)\right]-\left[A_{6,9}-\left(A_{8,9}+A_{10,9}+A_{12,9}+A_{14,9}\right)\right]\\ & -\left[A_{6,11}-\left(A_{8,11}+A_{10,11}+A_{12,11}+A_{14,11}\right)\right]-\left[A_{6,13}-\left(A_{8,13}+A_{10,13}+A_{12,13}+A_{14,13}\right)\right] \end{array}$$

(A17) $$\begin{array}{ll} B_{3,4} & =\left[A_{5,6}-\left(A_{7,6}+A_{9,6}+A_{11,6}+A_{13,6}\right)\right]-\left[A_{5,8}-\left(A_{7,8}+A_{9,8}+A_{11,8}+A_{13,8}\right)\right]-\left[A_{5,10}-\left(A_{7,10}+A_{9,10}+A_{11,10}+A_{13,10}\right)\right]\\ & -\left[A_{5,12}-\left(A_{7,12}+A_{9,12}+A_{11,12}+A_{13,12}\right)\right]-\left[A_{5,14}-\left(A_{7,14}+A_{9,14}+A_{11,14}+A_{13,14}\right)\right] \end{array}$$

(A18) $$\begin{array}{ll} B_{4,4} & =\left[A_{6,6}-\left(A_{8,6}+A_{10,6}+A_{12,6}+A_{14,6}\right)\right]-\left[A_{6,8}-\left(A_{8,8}+A_{10,8}+A_{12,8}+A_{14,8}\right)\right]-\left[A_{6,10}-\left(A_{8,10}+A_{10,10}+A_{12,10}+A_{14,10}\right)\right]\\ & -\left[A_{6,12}-\left(A_{8,12}+A_{10,12}+A_{12,12}+A_{14,12}\right)\right]-\left[A_{6,14}-\left(A_{8,14}+A_{10,14}+A_{12,14}+A_{14,14}\right)\right] \end{array}$$
